# Morphologically Cryptic Amphipod Species Are “Ecological Clones” at Regional but Not at Local Scale: A Case Study of Four *Niphargus* Species

**DOI:** 10.1371/journal.pone.0134384

**Published:** 2015-07-30

**Authors:** Žiga Fišer, Florian Altermatt, Valerija Zakšek, Tea Knapič, Cene Fišer

**Affiliations:** 1 Department of Biology, Biotechnical Faculty, University of Ljubljana, Ljubljana, Slovenia; 2 Eawag: Swiss Federal Institute of Aquatic Science and Technology, Department of Aquatic Ecology, Überlandstrasse 133, CH-8600 Dübendorf, Switzerland; 3 Institute of Evolutionary Biology and Environmental Studies, University of Zürich, Winterthurerstrasse 190, CH-8057 Zürich, Switzerland; 4 Slovenian Museum of Natural History, Ljubljana, Slovenia; Consiglio Nazionale delle Ricerche (CNR), ITALY

## Abstract

Recent studies indicate that morphologically cryptic species may be ecologically more different than would be predicted from their morphological similarity and phylogenetic relatedness. However, in biodiversity research it often remains unclear whether cryptic species should be treated as ecologically equivalent, or whether detected differences have ecological significance. In this study, we assessed the ecological equivalence of four morphologically cryptic species of the amphipod genus *Niphargus*. All species live in a small, isolated area on the Istrian Peninsula in the NW Balkans. The distributional ranges of the species are partially overlapping and all species are living in springs. We reconstructed their ecological niches using morphological traits related to feeding, bioclimatic niche envelope and species’ preference for epi-hypogean habitats. The ecological meaning of differences in niches was evaluated using distributional data and co-occurrence frequencies. We show that the species comprise two pairs of sister species. All species differ from each other and the degree of differentiation is not related to phylogenetic relatedness. Moreover, low co-occurrence frequencies in sympatric zones imply present or past interspecific competition. This pattern suggests that species are not differentiated enough to reduce interspecific competition, nor ecologically equivalent to co-exist via neutral dynamics. We tentatively conclude that the question of ecological equivalence relates to the scale of the study: at a fine scale, species’ differences may influence dynamics in a local community, whereas at the regional level these species likely play roughly similar ecological roles.

## Introduction

According to recent studies, morphologically cryptic species are a common and widespread phenomenon [1–2], found across all phyla and in different environments [3]. Although their detection has become routine [4–5], a common agreement on how cryptic species should be treated in biodiversity research is still lacking. At a global geographic scale, our ignorance of cryptic species leads to underestimations of species richness [6], while their discovery may modify hypotheses of their ranges [7–8]. At a regional and local scale, the presence of cryptic species might affect our view on the local occurrence of species, their interactions with coexisting species and their response to the local abiotic environment. Furthermore, our awareness and understanding of morphologically cryptic species may profoundly affect their conservation status and priority setting of endangered species.

All these issues are critically related to a question of whether or not morphologically cryptic species are ecologically equivalent. If equivalent, such species could be considered as “ecological clones”, playing a highly predictable role in the ecosystem across different scales. Several lines of evidence suggest that morphologically cryptic species indeed share their ecological needs. First, morphology itself is related to species’ ecological niche [9]. Outstanding morphological similarity of cryptic species is maintained by stabilizing selection [10], which implies that such species exploit the same microhabitat and resources. Moreover, many cryptic species are sister species deriving from the same ancestor (but see [11]) and might have retained the ancestral ecological niche [12–13]. On the other hand, some studies found evidence for divergence of morphologically cryptic species in aspects of the ecological niche unrelated to morphology [14–16].

Therefore, a key question related to evolutionary ecology of cryptic species is how phylogenetic differentiation translates into ecological differentiation. An accurate answer requires an experimental approach that is often time consuming and sometimes even unfeasible due to extreme morphological crypsis of these animals. Considerable insights, however, can be already gained from comparative analysis of ecological and distributional data in well-defined regions. On rare occasions, morphologically cryptic species come in contact with each other and establish zones of sympatry [14, 17–18]. Such cases may be particularly promising for exploring the ecological divergence among cryptic species, as species in sympatry experience intensified interspecific competition [9] that may drive divergent evolution of the components of the ecological niche that are not under stabilizing selection [19] and maximize their ecological difference.

Here, we present a case study, in which we explored the extent of ecological differentiation of four morphologically cryptic species belonging to the subterranean amphipod genus *Niphargus*. The system is a species-poor community comprising of two nonrelated, but morphologically and ecologically highly similar lineages that had long been treated as a single species [20]. Each lineage (the *N*. *krameri* complex and the *N*. *spinulifemur* complex, see [11]), however, each comprises two morphologically cryptic species. All species live in a small region, the Istrian Peninsula (ca. 2,800 km^2^) in NW Balkans [21]. The study area has been isolated from the rest of Dinaric Mountains for several millions of years [22]. The ecology of the focal species strongly suggests that these species have never occurred beyond this area [21], and that processes of ecological differentiation as well as species interactions are confined to this geographic area. The four species share general ecological requirements, and can all be found predominately in springs, which are species-poor, predator-free boundary environments between surface and groundwater systems. Thereby, this natural study system is an ideal, diversity-balanced system of sympatric and partially sympatric cryptic species: it is spatially well defined and simple enough to infer species interactions based on co-occurrence data, while still complex and diverse enough to explore the impact of phylogeny and ecological differentiation.

In this study we first determined the focal species using molecular data. Next, we explored their ecological properties, using type of habitat, bioclimatic requirements and morphological traits related to feeding biology. To evaluate ecological significance of between-species differences we tested for signs of competition among these species based on co-occurrence data. We show that the studied cryptic species are ecologically differentiated; however, co-occurrence data suggest that this differentiation is not sufficient to resolve the possible interspecific competition at the local scale. While interspecific competition likely contributes to local community dynamics, the incomplete differentiation suggests that these species likely play a more or less similar role in the ecosystem at a regional scale.

## Materials and Methods

### Data

Data on *Niphargus* occurrences on the Istrian Peninsula were obtained from the Zoological Collection of the Department of Biology (Biotechnical Faculty, University of Ljubljana) and previously published papers. The entire peninsula and its surrounding area have been systematically explored over past ten years, with special attention to caves, springs and uppers stretches of surface streams. Part of the data has been already published [21], but many localities were visited for the first time and many sites were examined several times.

Some of the samples (collected between March 2002 and October 2011) were used to resolve the taxonomy of both species complexes using molecular data (details about samples in S1 Table). Samples from the other localities could be unambiguously assigned to the newly delimited species. All the data was assembled into an original dataset of 238 unique localities (S2 Table). It contained 31, 73, 6 and 150 locality records of *N*. *krameri A*, *N*. *krameri B*, *N*. *spinulifemur* A and *N*. *spinulifemur B*, respectively. The original dataset was visually inspected using DIVA-GIS 7.5.0 (http://www.diva-gis.org) and each record was assigned to a grid with cell size of 3 x 3 km. This produced a dataset with 25, 48, 6 and 65 presence records of *N*. *krameri A*, *N*. *krameri B*, *N*. *spinulifemur* A and *N*. *spinulifemur B*, respectively (S3 Table). Unless stated differently, this dataset was used for spatial and ecological analyses.

**Permits and approvals** were not required for sampling at these locations. The study does not include endangered or protected species.

### Description of the study system and taxa

The study focused on species of the *N*. *krameri* and the *N*. *spinulifemur* species complexes, both inhabiting the Istrian Peninsula. Two distinct phylogenetic lineages are found within each, in both cases called A and B respectively (see Results). Other amphipods living in the region and occasionally found at the sampled localities were excluded from analyses as they live in completely different habitats and were only recorded as by-catches. These included other *Niphargus* species from deep cave lakes and coastal anchihaline caves [23] and *Gammarus* and *Echinogammarus* amphipods that live only in permanently watered streams [21,24]. Focal species were found predominantly in springs and upper stretches of streams, but also in caves. All these habitats are species poor: aside from amphipod fauna, assellote isopods, trichopteran, plecopteran and ephemeropteran larvae, erpobdelid leeches and salamander larvae were also found, but no fishes were observed. The effects of predation are likely negligible. Juvenile amphipods of focal species shelter in fine substrate [24]. Adults are too fast and too large (>15 *mm*) to be predated by leeches or by salamander larvae.

### Phylogenetic characterization of the community

The phylogeny of the *N*. *krameri* and *N*. *spinulifemur* species complexes was established using molecular data. We employed two criteria for species delimitation: i) monophyly and ii) genetic distance measured as patristic distance [25].

Species complexes were identified according to diagnostic characters [20–21]. After the morphological examination, DNA was isolated either from one pereopod (large specimens) or whole abdomen (small specimens) using the GeneElute Mammalian Genomic DNA Miniprep Kit (Sigma-Aldrich) following the protocol for Mammalian Tissue Preparation. We amplified a nuclear gene coding for the 28S ribosomal subunit (28S rRNA) and a fragment of the mitochondrial gene coding for the cytochrome oxidase I (COI). The nuclear marker was amplified using primers 28S lev3 and 28S des5 (1268–1382 bp; [26–27]). The mitochondrial marker COI was amplified using primers Jerry and Maggie (592bp, [28]) for *N*. *krameri*. As amplification with those primers was not successful in *N*. *spinulifemur* specimens, we used either LCO 1490 and HCO 2198 (661 bp; [29]) or the newly designed COI_spf1 (5′- GNACCTTATATTTTATTTTAG -3′) and COI_spr1 (5′- CGRTCTGTTARTAATATWGTAAT -3′) primers (548 bp) for specimens that gave no PCR products with the former as well. Although the COI fragments obtained for each species are not homologs and do not overlap, it has been tested that in *Niphargus* and cave shrimp *Troglocaris* they exhibit the same phylogenetic information (C. Douady unpublished, V. Zakšek unpublished).

PCR was performed using the next cycling settings: 45 s at 94°C, 30 s at 48°C, 60 s at 72°C for 40 cycles followed by final extension of 7 min at 72°C for the 28S rRNA (28S lev3 and 28S des5); 60 s at 95°C, 60 s at 46°C, 90 s at 72°C for 45 cycles followed by final extension of 7 min at 72°C for the COI (Jerry and Maggie) and 60 s at 94°C, 60 s at 45°C, 150 s at 72°C for 40 cycles followed by final extension of 7 min at 72°C for the COI (LCO 1490 and HCO 2198; COI_spf1 and COI_spr1).

PCR products were purified using Multiscreen PCR plates (Millipore) according to the manufacturer’s instructions. Many reactions with 28S rRNA primers gave unspecific products. The target product was excised from a 1.5% agarose gel and purified using GeneJET Gel Extraction Kit (Fermentas) following the manufacturer’s protocol. Each fragment was sequenced in both directions using appropriate PCR amplification primers (Macrogen). Contigs were assembled and edited using Geneious 6.0.5. (Biomatters). Sequences of five distantly related *Niphargus* species, used as outgroup taxa, were obtained from GenBank and our database (for details see S1 Table).

Four alignments, separated by species and gene, were made using MAFFT 7.017 [30] plug-in in Geneious 6.0.5. (Biomatters). Each species alignment was analysed as a four partition dataset (28S, COI 1st codon position, COI 2nd codon position, COI 3rd codon position) by Bayesian inference using Mr. Bayes 3.2.1 [31]. The appropriate substitution model and priors were chosen for each partition according to BIC criterion computed with jModelTest 2.1.4 [32–33]. Two independent runs with four chains were run for 2 × 10^6^ generations and sampled every 100 generations. This was sufficient for the SD of split frequencies to drop under 0.01. After discarding the first 25% of the sampled trees from both runs, a 50% majority rule consensus tree was constructed from remaining 30000 trees. Additionally, we searched for the tree topology with the maximum likelihood using PHYML 3.1 [33]. The robustness of the topology was tested with 1000 bootstrap replicates. Finally, the maximum likelihood tree, without bootstraps, based only on COI alignment was searched for in both species. Patristic distances were extracted from these trees using Patristic [34] and average patristic distances were computed between clades. We also calculated average uncorrected p-distances and Kimura two-parameter distances (K2P) between clades based on COI sequences using MEGA 6 [35].

### Ecological niche characterization

Three independent axes of the ecological niche were explored: morphological traits important for feeding biology, epi-hypogean distribution of species and bioclimatic niche. Each axis was given equal weight in the three-dimensional niche model.

#### Morphology

Amphipods may feed on decaying leaves, filter organic particles or predate [36]. Any specialization in feeding biology might represent a critical shift in ecological niche and stabilize species coexistence. Appendages involved in feeding include mouthparts and gnathopods. The four species were found to differ in three such morphological characters dactylus of maxilliped, ischium of gnathopod I, and carpus of gnathopod II (for an overview of niphargid morphology see [37]). The number of maxilliped’s claws presumably enhances the grip on organic particles whereas extra setal groups on gnathopods are thought to enlarge the capacity of a filtering basket between the gnathopods and the body. Traits are non-polymorphic in molecularly delineated species. For discrimination of feeding ecology on a continuum between predation-filtration, all three traits were treated as equally important. Each trait was treated as a binary variable and each species was assigned an index based on these three binary characters in a range between 0 (predator) and 3 (filtrator). The difference in feeding ecology was estimated by pairwise comparison with 3 and 0 attributed to the most and the least different species pair, respectively. For the joint comparison of three-dimensional niche, differences were normalized to a range between 0 and 1. Body size, a trait commonly used as a surrogate for ecological niche, turned out to be non-informative.

#### Epi-hypogean distribution

*Niphargus* species are adapted to subterranean environment (eyeless, little or no protective pigment [37]). However, species in this study are most commonly found in karstic springs and, to a lesser extent, in caves and upper stretches of streams that dry out during the summer. The distribution along the epi-hypogean water gradient represented the second niche axis. A habitat type was assigned to localities of the original dataset based on the locality descriptions; 13 localities were excluded as their descriptions were uninformative. Three habitat type categories, namely ‘surface’ (streams, small rivers, puddles, swamps, water supplies and reservoirs), ‘transitional’ (natural or artificial springs and sinks) and ‘subterranean’ (caves, artificial tunnels, aqueducts and mines) were distinguished. Istrian Peninsula is geologically diverse. Although geological basement does not affect the distribution of species [21] it may affect the distribution of available habitats. To control for the impact of geology, we assigned geological basement (flysch or limestone) to each locality according to the georeferenced geological map of the area [38–44]. A three-way contingency table (species, habitat type, geology) was constructed and conditional independence of species and habitat type given geology was estimated with Cochran-Mantel-Haenszel chi-squared test statistic. Due to low expected frequencies in some cells, a randomization test with 10.000 table permutations was performed in R 3.0.1. (R Core Team 2013, URL http://www.R-project.org/).

#### Bioclimatic niche

Compared to epigean environments, subterranean environments are relatively stable. The temperature in a cave corresponds to the mean annual temperature of its epigean locality and many subterranean species are indeed very sensitive to temperature fluctuations [45]. Temperature fluctuations can be considered as surrogates for environmental variation [46]. In addition, precipitation regime may cause catastrophic ecological drift and can directly relate to temporal instability of habitat template [47]. Given that species may differ in their bioclimatic niche, potential species distributions (i.e., bioclimatic niche envelopes) were modelled using MaxEnt 3.3.3e (http://www.cs.princeton.edu/~schapire/maxent; [48]), which has been reported to work well with small sample sizes [49] and successfully predicts non-colonized habitat patches that are suitable for the focal species. It is also not sensitive to possible effects of interspecific interactions [48].

The modelled area was the Istrian Peninsula. Original WorldClim climatic layers [50] were resampled to a cell size of 3x3 km. To account for non-independence of BioClim data, we computed Spearman`s rank correlation coefficients across all pairs of variables. Three correlation coefficient values (0.7, 0.8, 0.9) were used as threshold values beyond which climatic data were assumed independent. This procedure yielded three different model inputs counting four, five and eight different BioClim variables (Table 1). Each of them was run for each species. Auto-feature mode and settings suggested by [48] were used. Models were trained using 75% of presence points and tested against the remaining data. Jackknife was used to train distribution models of *N*. *spinulifemur* A, as the small number of records makes the above method inappropriate [49]. Predictive accuracy of all models was evaluated with area under receiver operating characteristic curve (ROC-AUC) statistics.

**Table 1:** BioClim variables used in modelling the bioclimatic niches.

Variable Code	Variable	0.9	0.8	0.7
Bio 1	Annual Mean Temperature	+	-	-
Bio 3	Isothermality*	+	+	+
Bio 4	Temperature Seasonality (standard deviation *100)	+	+	+
Bio 7	Temperature Annual Range	+	-	-
Bio 9	Mean Temperature of Driest Quarter	+	-	-
Bio 12	Annual Precipitation	+	+	+
Bio13	Precipitation of Wettest Month	+	-	-
Bio 15	Precipitation Seasonality (Coefficient of Variation)	+	+	+
Bio18	Precipitation of Warmest Quarter	+	+	-

0.7, 0.8, 0.9 refer to threshold of the maximum correlation (Spearman’s rank) between BioClim variables value beyond which the data were considered as independent.

*Isothermality = [Mean Diurnal Range (Mean of monthly (max temp–min temp)) / Temperature Annual Range]

To compare the bioclimatic niche envelopes of four studied species, functions in dismo [51] and phyloclim [52] R package were used. Pairwise niche envelope overlap was estimated from continuous probability maps (we used mean values of six alternative maps) with Schoener’s *D* index [53]. This metrics ranges from 0 (no overlap) to 1 (identical potential distributions). The significance of indices was tested using a niche equivalency test, which asks whether the bioclimatic niche envelope models of two species are more different than expected if they were randomly drawn from the same underlying distribution [53].

#### Joint, three-dimensional niche model

To estimate the ecological differences between focal species, a three-dimensional niche model was constructed. A difference of a species pair in one niche aspect was represented along each axis (feeding biology, epi-hypogean distribution, bioclimatic niche). Morphology was treated as described above. Habitat type was represented as a difference in proportion of surface *versus* subterranean habitats (transitional habitats were excluded, see Results). Schoener’s D index was used as a measure of difference in bioclimatic niches. Joint niche differences were calculated as magnitudes of vectors defined by a species pair. After normalization, values along each niche axis ranged between 0 (maximum difference) and 1 (maximum similarity). Three estimates were obtained for every species-pair as three values of Schoener’s D index were available for each.

### Inferring ecological equivalence from distributional data and coexistence models

#### Distribution pattern tests

To test whether species show random, evenly dispersed or clumped distribution a nearest neighbour analysis was performed using Spatial Statistics toolbox in ArcMap 10.0 (ESRI 2011).

#### Tests of competition

Competition tests were made on assumptions and premises as follows. If interspecific species competition does not exceed intraspecific competition [54], the presence of one species has no influence on the presence of the other. Therefore, the two species’ occurrences can be treated as independent events and the chance of finding both species in syntopy equals to the joint probability of independent events, that is, a product of the probabilities of finding each species in a given region.

When species are spatially segregated and ecologically at least slightly different, it is difficult to tell apart to what extent spatial segregation can be attributed to differences in niches (e.g., bioclimatic niche envelope) of studied species and to what extent this segregation can be considered as the result of competitive interaction. For this reason, we inferred the role of competition only in areas where neither geographical distances nor ecological conditions presumably restrict dispersal. As climate factors potentially affect species’ distribution, the analysis was constrained to the areas where species come in contact according to bioclimatic niche envelope predictions.

Similar reasoning applies to spatial segregation along surface-subterranean niche axis. To account for this, we made another even more restrictive analysis, in which we included the data from those localities where subterranean and surface species may come in contact with each other, i.e., in springs. The second analysis is therefore limited to localities from springs found in zones of sympatry.

Continuous probability maps were converted to presence-absence maps in ArcMap 10.0 (ESRI 2011) using minimum training presence (LPT) computed in MaxEnt as a threshold. Consequently, three alternative binary distribution maps were obtained for each species (S1–S4 Figs).

Using all alternative binary distributions, all possible areas of pairwise sympatries were identified. The probabilities of occurrences of each species and the probabilities of syntopies were estimated using i) the original data, and alternatively, ii) using only records from springs. The probabilities of occurrences of each species and the probabilities of syntopies served as a null hypothesis of no competition that was tested against the real data with an exact multinomial test in EMT R package [55].

Altogether six tests were performed for all species pairs. Three tests were controlling for the effect of climate factors and three tests were controlling for the effect of climate factors and for the effects of epi-hypogean distribution.

## Results

### Evidence for speciation

**Fig 1:**
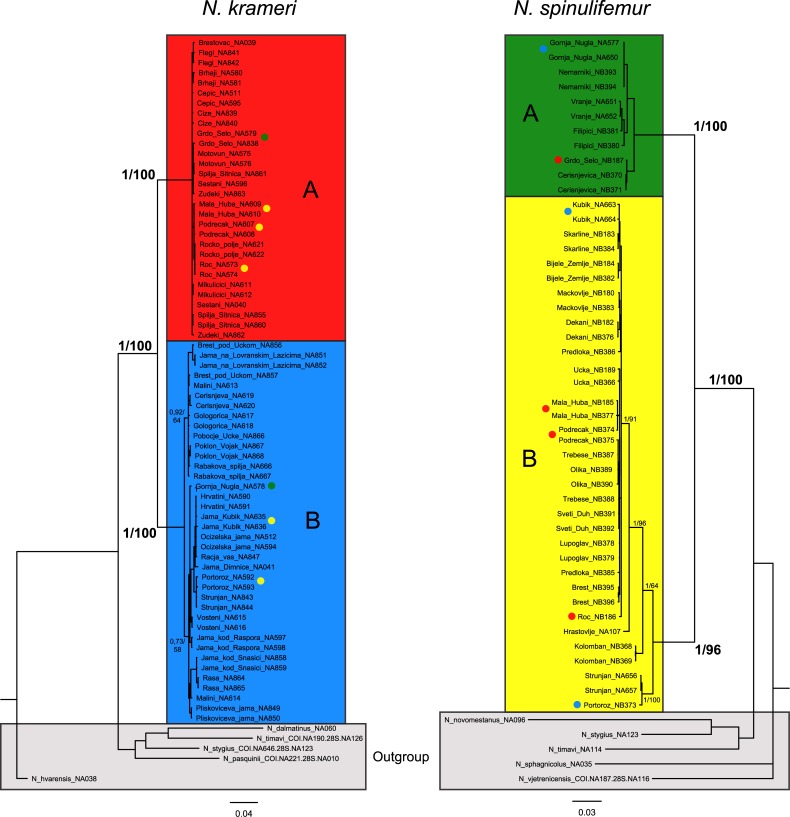
Bayesian phylogenetic trees of the focal species complexes using concatenated alignments for 28S and COI gene fragment. The numbers on nodes indicate posterior probabilities for Bayesian trees and bootstrap support values for maximum likelihood trees. Patristic, K2P and uncorrected p-distances between A and B species within *N*. *krameri* and *N*. *spinulifemur* are 0.34, 0.14, 0.12 and 0.36, 0.19, 0.16, respectively. Coloured dots at some terminals indicate localities of co-occurrences with the respective species. The species are: *Niphargus krameri* A (NKA, red), *Niphargus krameri* B (NKB, blue), *Niphargus spinulifemur* A (NSA, green), and *Niphargus spinulifemur* B (NSB, yellow).

**Fig 2:**
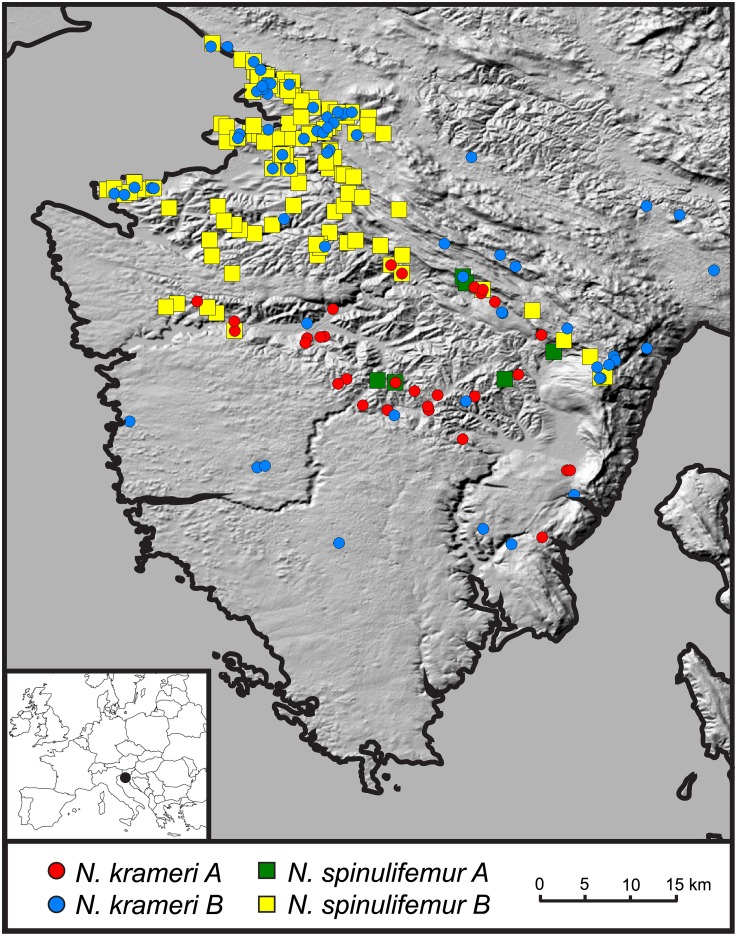
Distribution of focal species and the studied area (the Istrian Peninsula). The inset map indicates the geographic position of the study area within Europe. Species presence records were superimposed on a SRTM Shaded Relief (Central North) layer available from ESRI.

Both *N*. *krameri* and *N*. *spinulifemur* are monophyletic complexes (Fig 1, see also [11]). Each complex is further partitioned into two clades, which are labelled clade A and B in both cases. These clades are strongly supported by Bayesian inference as well as maximum likelihood methods. The average patristic distances between clades calculated from COI maximum likelihood trees by far exceed 0.16 substitutions per nucleotide (Fig 1), which has been proposed as a conservative threshold for delimiting species in crustaceans [25]. The two clades within the *N*. *krameri* complex can be distinguished by the presence or absence of a single obscure character, an additional setal group on carpal article of gnathopod II (Table 2). No diagnostic character was found between the clades of the *N*. *spinulifemur* complex, despite the investigation of a long list of potential characters (for an overview see [37]). Some species were found to co-occur (Figs 1 and 2). The possibility of zones of sympatry between clades strongly suggests their mutual reproductive isolation. Considering the presented facts, we argue that the four clades represent four reproductively isolated and morphologically cryptic species, namely *N*. *krameri A*, *N*. *krameri B*, *N*. *spinulifemur A* and *N*. *spinulifemur B*. Respective name abbreviations NKA, NKB, NSA and NSB are used throughout the text. Formal description of the new species will be published in another paper.

**Table 2:** Species`differences in morphological traits related to feeding biology.

Morphological trait	*N*. *krameri* A	*N*. *krameri* B	*N*. *spinulifemur* A	*N*. *spinulifemur* B
maxilliped dactylus	double nail	double nail	single nail	single nail
carpal article of gnathopod II, extra setae forming filtering basket*	well developed	missing	well developed	well developed
ischium I, extra setae forming filtering basket	missing	missing	well developed	well developed

*diagnostic character between *N*. *krameri* A and *N*. *krameri* B.

As species delimitation may be sensitive to the choice of method (see e.g., [56]), we additionally, and as a part of a more extensive analysis on the whole genus *Niphargus*, confirmed our species delimitations using PTP and bPTP approaches (Fišer et al. unpublished work). These gave identical delimitations for the *N*. *krameri* species complex and split *N*. *spinulifemur* A and B into a few additional species. Because accurate species delimitation requires a multilocus approach comprising two or more independent markers [56], and as some of the results of the PTP and bPTP methods are discussable (work in preparation), we decided to go with the well supported, robust and most conservative estimates obtained by Bayesian inference and maximum likelihood.

### Ecological niche of the four species

The four study species differ both along individual niche axes as well as in their three-dimensional ecological niche. The differences among species are of different magnitude. The results are summarized below.

Morphology of mouthparts and gnathopods suggests that the most different species are NKB *versus* NSA and NSB. The former species seems to be oriented towards predation and/or handling large particles of food, whereas the latter two species are likely specialized in filter-feeding. Small differences in morphology were found between NKA (filter feeder/predator) and NKB (predator), and no differences were found between NSA and NSB (both of them filter-feeders, Table 2).

**Fig 3:**
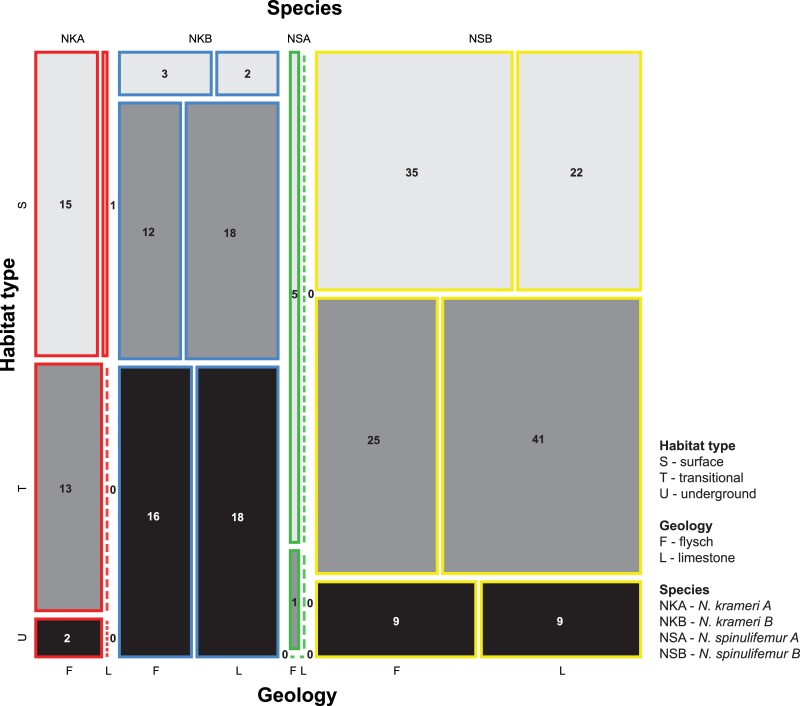
Epi-hypogean distribution of the studied species, corrected for geological basement.

The analysis of epi-hypogean distribution indicates that about one half of records for each species derive from transitional habitats. NKA, NSA and NSB, however, are more common in surface habitats whereas NKB inhabits subterranean habitats more frequently (M^2^ = 53.8, *p* = 0.001; Fig 3). Geological basement does not affect the observed, non-random partitioning of habitat type among species.

All models defining the bioclimatic niche envelope can be considered as acceptably predictive (AUC > above 0.7, see [57]). Details of each model can be found in (S1–S4 Figs). Niche overlaps in terms of Schoener’s *D* ranged from 0.38 to 0.81 and are listed in Table 3. Disregarding which combination of bioclimatic variables was used to model the bioclimatic niche envelope, the indices reveal almost identical results. The overlap of niches is moderate (0.4–0.6) to high (0.6–0.8) [58]. Niche equivalency test revealed that bioclimatic niches are equivalent within NKA and NSA as well as within NKB and NSA. In the remaining species pairs, bioclimatic niches are significantly more different than expected if they were randomly drawn from the same underlying distribution. However, different accuracy of niche models advises some care, as the overlap estimates are subjected to unequal errors. Error is smallest between NKA and NSB and greatest between NKB and NSA.

**Table 3:** Ecological similarity between species pairs along individual niche axes and the joint niche.

Species pair	Feeding biology^1^	Epi-hypogen distr.^2^	Bio Clime^0.7^	Bio Clime^0.8^	Bio Clime^0.9^	Joint niche^0.7^	Joint niche^0.8^	Joint niche^0.9^
**NKA-NKB**	0.67	0.24	0.63*	0.69*	0.60*	0.55	0.57	0.54
**NKA-NSA**	0.33	0.89	0.70	0.74	0.75	**0.80**	**0.80**	**0.80**
NKA-NSB	0.33	0.87	0.41*	0.41*	0.38*	0.59	0.59	0.58
NKB-NSA	0.00	0.13	0.77	0.81	0.75	0.58	0.58	0.58
**NKB-NSB**	0.00	0.37	0.64*	0.58*	0.62*	**0.43**	**0.40**	**0.41**
NSA-NSB	1.00	0.76	0.45*	0.45*	0.44*	0.77	0.77	0.77

^1^Overall morphological similarity calculated from Table 2, normalized values.

^2^Difference in preference for surface habitats, normalized values.

^0.7, 0.8, 0.9^ The values denote maximum Spearman’s rank correlation between BioClim variables allowed in calculation of Schoener’s D index. For calculations of the joint niche values of D index were standardized.

*A statistically significant difference in values of selected BioClim variables for the species pair.

Overall differences between species inferred from our three-dimensional niche model are presented in Table 3. Three alternatives of bioclimatic niche have a negligible effect on the final values. Most similar niches are shared by NKA-NSA and NSA-NSB species pairs whereas niches between NKB and NSB are most different. Other species pairs lie in between these extremes. It is important to notice that the two species pairs that appear most sympatric in Fig 2 (NKA-NSA and NKB-NSB) exhibit the most and the least similar niche. Likewise, niches between sister species pairs are either similar (NSA-NSB) or quite different (NKA-NKB).

### Coexistence models in the community

The four species exhibit a sympatric distribution pattern across the Istrian Peninsula (Fig 2). The nearest neighbour analysis further demonstrated that the distribution of each species is clumped rather than randomly or evenly dispersed (Table 4). Apparently, the distribution overlap is greatest in phylogenetically distantly related (NKA-NSA and NKB-NSB) rather than in sister species (NKA-NKB and NSA-NSB) (Fig 2 and S1–S4 Figs).

**Table 4:** Evidence for clumped distribution of focal species.

Species	N	Area	NN Ratio*	*z*-score	*p*-value
NKA	25	0.51	0.47	-5.0444	<0.0001
NKB	48	0.51	0.82	-2.4182	0.0156
NSA	6	0.51	0.23	-3.5940	0.0003
NSB	65	0.51	0.51	-7.4832	<0.0001

* NN Ratio = observed mean distance/expected mean distance, if NNR<1 then distribution is clumped, if NNR>1 then distribution is equally dispersed.

Observed and expected values of syntopic and non-syntopic species-occurrences in areas of sympatry (see Methods) along with results of multinomial tests are presented in Table 5 and S4 and S5 Tables. Six alternative tests (see Methods and Table 5) were performed for each species pair. Overall, in five out of six species pairs it seems that syntopies were less frequent and non-syntopic occurrences were more frequent than would be expected by chance alone in all tests.

**Table 5:** Evidence for competition inferred from presence-absence distributions.

species pair^1^	corrected for bioclimatic niche envelope^2^			corrected for bioclimatic niche envelope and epi-hypogean spatial seggregation^3^		
	0.7^4^	0.8^4^	0.9^4^	0.7^4^	0.8^4^	0.9^4^
NKA—NKB	**<0.001** ^5^	**<0.001**	**<0.001**	**0.007**	**<0.001**	**0.009**
NSA—NSB	0.12	0.12	0.534	0.12	0.12	0.534
NKA—NSA	**<0.001**	**<0.001**	0.055	**0.004**	**0.008**	0.055
NKB—NSB	**<0.001**	**<0.001**	**<0.001**	**<0.001**	**<0.001**	**<0.001**
NKA—NSB	**0.034**	**<0.001**	0.249	**0.034**	**<0.001**	0.249
NKB—NSA	**0.011**	**0.027**	0.099	0.23	0.23	NA^6^

^1^ NKA, NKB, NSA and NSB abbreviate the four species of the NK and NS complex.

^2^ Sympatry area of a species pair defined as overlapping ranges, as inferred by LPT binary threshold.

^3^ Sympatry of species pair in area of overlapping ranges as inferred by LPT binary threshold and corrected for epi-hypogean spatial segregation (data from springs only).

^4^ Correlation threshold defining the BioClim variables used in modeling (see Table 1).

^5^ Probability that the observed frequencies of co-occurrence come from the same underlying distributions as the expected frequencies. Boldface values indicate statistical significance for nonrandom low co-occurrence frequency, which might imply present or past competitive interactions. More detailed tables including expected and observed number of occurrences and M statistics are available in Supporting Information.

^6^ Controlling for spatial autocorrelation restricted the number of records and probability could not have been estimated.

In NKA-NKB and NKB-NSB species pairs, syntopies were less frequent and non-syntopic occurrences were more frequent than would be expected by chance alone in all tests. The same is true in four out of six tests for NKA-NSA and NKA-NSB (Table 5 and S4 and S5 Tables). In NKB-NSA species pair increasing restrictions yielded in five tests alone; syntopies were less frequent and non-syntopic occurrences were more frequent than would be expected by chance alone in two out of five tests. The only species pair that in no test differed from chance expectations was NSA-NSB. We tentatively propose that the pattern implies competitive interactions within four to five out of six pairs of species. All results including phylogenetic relatedness, niche differentiation, distribution and tests of co-occurrence are summarized in Table 6.

**Table 6:** Summary of all results.

Species pair	Phylogenetic relatedness	Ecological niche^1^	Distribution pattern^2^	Competition
NKA—NKB	sister	5	small spatial overlap	strong
NSA—NSB	sister	2	small spatial overlap	unlikely
NKA—NSA	non- sister	1	strong spatial overlap	strong
NKB—NSB	non- sister	6	strong spatial overlap	strong
NKA—NSB	non- sister	3	small spatial overlap	strong
NKB—NSA	non- sister	4	small spatial overlap	?

^1^ Numbers rang species pairs according to the similarity of the species ecological niche: 1- little difference, 6—maximum differences.

^2^ All species exhibit a clumped distribution (see Table 4).

## Discussion

### The significance of ecological differentiation

All four focal species ecologically differ from each other and the degree of differentiation varies between species (Table 6). These differences may not necessarily mean that species play ecologically different roles in the ecosystem. For example, the four species studied here are spatially segregated. Such segregation may indicate species’ adaptation to for example bioclimatic niches, but it may also be a result of biotic interactions [59–60]. In order to evaluate whether the observed differences indicate species’ ecological differentiation, we analysed ecological differences and co-occurrence data within coexistence theory.

The most differentiated species pair in the system is NKB-NSB (Tables 3 and 6). Strongly differentiated species are expected to establish stable coexistence, defined as long-term co-occurrence where intraspecific competition exceeds interspecific competition [54], and where each species shows positive population growth when it is rare in the system [61]. If these criteria were satisfied, co-occurrence records should be more frequent than observed by chance, given that in NSB the geographic range is spatially nested within the range of NKB. Apparently this is not the case as the frequency of co-occurrences is unexpectedly low. A similar pattern with unexpectedly low co-occurrences might occur if species were specialized for spatially auto-correlated environmental factors that would limit their dispersal. In the present study, however, we controlled for bioclimatic factors and surface-subterranean segregation by limiting our inference to sympatric areas, hence the effect of restricted dispersal between localities should be minimal. Although alternative explanations cannot be excluded without experimental work, we tentatively propose that this pattern hints at short-term co-occurrences and competitive exclusion [62]. In other words, ecological differences between these two species are not sufficient to resolve interspecific competitive interactions.

In analogy, ecologically equivalent species, which might evolve under various different theoretical predictions [63–64], could establish prolonged periods of co-occurrences (unstable coexistence *sensu* [54]). The ecologically most similar pair in the study system is NKA-NSA. Distributional ranges of both species overlap and both species apparently share bioclimatic and epi-hypogean requirements. The only difference observed was in morphology, and even in this aspect NKA shows a degree of similarity with NSA, possibly indicating NKA convergence towards ecological equivalence. Ecologically equivalent species are expected to co-occur with a frequency that equals the probability of chance meetings. Again, we found a different pattern: although the two species spatially overlap, no co-occurrences between NKA and NSA were detected. If not result of low number of records for NSA, this result again implies competitive exclusion [62].

Other species pairs (NKA-NKB, NKA-NSB, NKB-NSA, NSA-NSB) comprise of moderately different species, the ranges of which spatially overlap only in part. Spatial segregation may indicate an adaptation to spatially-correlated environmental parameters like bioclimatic niche. In turn, dispersal along an environmental gradient establishes zones of sympatry, where species interact with each other [65]. In most cases, frequency of co-occurrences is low (Table 5 and S4 and S5 Tables) which once again indicates that interspecific interactions do not allow long-term co-occurrences in zones of sympatry. The only exception is the species pair NSA-NSB, where results suggest that these two species may co-occur at random. However, the zone of sympatry is small. Consequently the number of records used in co-occurrence test in this species pair is low, and results may be inaccurate.

In short, we tentatively conclude that all four studied species are neither ecologically identical, nor functionally differentiated. Results may indicate that any pair of the four studied species experience strong interspecific interactions at local-most scale, or, with other words, the observed ecological differentiation should be considered when dynamics of local community is studied. Noteworthy, these results are concordant to recently published experimental approach conducted with cryptic amphipods from the genus *Hyalella* [66].

### Phylogenetic differentiation does not relate to ecological differences

Several authors recently addressed whether phylogenetic relationship can be used as a proxy of species’ ecological similarity [13, 67–69]. Here presented results do not support the hypothesis that closely related species are ecologically more similar than distantly related ones: species of *N*. *spinulifemur* complex are among the most similar species in the studied system, whereas species of *N*. *krameri* complex are among the most different herein studied species pairs (Table 6). We acknowledge that the dataset is too small to permit statistical evaluation of this hypothesis. In addition, generalization of this conclusion may be sensitive to phylogenetic hierarchy. Wiens [12] argued that the concept of ecological similarity applies well to clade level, although species within the clade differ from each other. Our data may be in agreement to this view: all species differ from each other; however, both species complexes studied share similar ecology that is distinct from ecology of many other *Niphargus* clades [5, 27].

## Conclusion

The question of ecological differentiation of morphologically cryptic species is apparently a hierarchy-related question, no matter if considered from phylogenetic or functional-ecological perspective. Species differ at a fine scale and this differentiation may play a role in local dynamics, for example processes involved in community assembly [66, 70–72]. We strongly advise that morphologically cryptic species should not be *a priori* treated as ecologically equivalent in fine scale ecological studies or even eco-toxicological tests [73–75]. By contrast, incomplete differentiation as inferred from low-occurrences implies that on a large scale these species play roughly similar roles in the ecosystem and that clade membership can be used as a proxy for species ecology.

## Supporting Information

S1 Fig Estimated distribution of *Niphargus krameri* A. (DOC)

S2 Fig Estimated distribution of *Niphargus krameri* B. (DOC)

S3 Fig Estimated distribution of *Niphargus spinulifemur* A. (DOC)

S4 Fig Estimated distribution of *Niphargus spinulifemur* B. (DOC)

S1 Table List of samples used in phylogenetic analyses with their locations and GenBank accession numbers of their sequences. (XLSX)

S2 Table Original dataset of 238 unique localities with assigned geology and habitat type values. (XLSX)

S3 Table Dataset of 144 unique localities with values of the 19 BioClim variables. (XLSX)

S4 Table Evidence for competition inferred from presence-absence distributions corrected for bioclimatic niche envelope (all data presented). (DOC)

S5 Table Evidence for competition inferred from presence-absence distributions corrected for bioclimatic niche envelope and epi-hypogean spatial segregation (all data presented). (DOC)
