# Supplementary material for: Morphologically Cryptic Amphipod Species Are “Ecological Clones” at Regional but Not at Local Scale: A Case Study of Four Niphargus Species
Source: PLoS One. 2015 Jul 30;10(7):e0134384. doi: 10.1371/journal.pone.0134384 (PMC4520478; doi:10.1371/journal.pone.0134384)
Supplement: S1 Fig — (DOC) [file pone.0134384.s001.doc]

**Morphologically cryptic amphipod species are “ecological clones” at regional but not at local scale: a case study of four *Niphargus* species**

**Supporting Information S6-9 Figures**

Žiga Fišer^1^, Florian Altermatt^2,3^, Valerija Zakšek^1^, Teja Knapič^4^, Cene Fišer^1^

^1^Department of Biology, Biotechnical Faculty, University of Ljubljana; Večna pot 111, SI-1001, Ljubljana, Slovenija.

^2^Department of Aquatic Ecology, Eawag: Swiss Federal Institute of Aquatic Science and Technology, Überlandstrasse 133, CH-8600 Dübendorf, Switzerland.

^3^Institute of Evolutionary Biology and Environmental Studies, University of Zurich

Winterthurerstr. 190, CH-8057 Zürich, Switzerland.

^4^Slovenian Museum of Natural History, Prešernova 20, SI - 1001 Ljubljana, Slovenija.


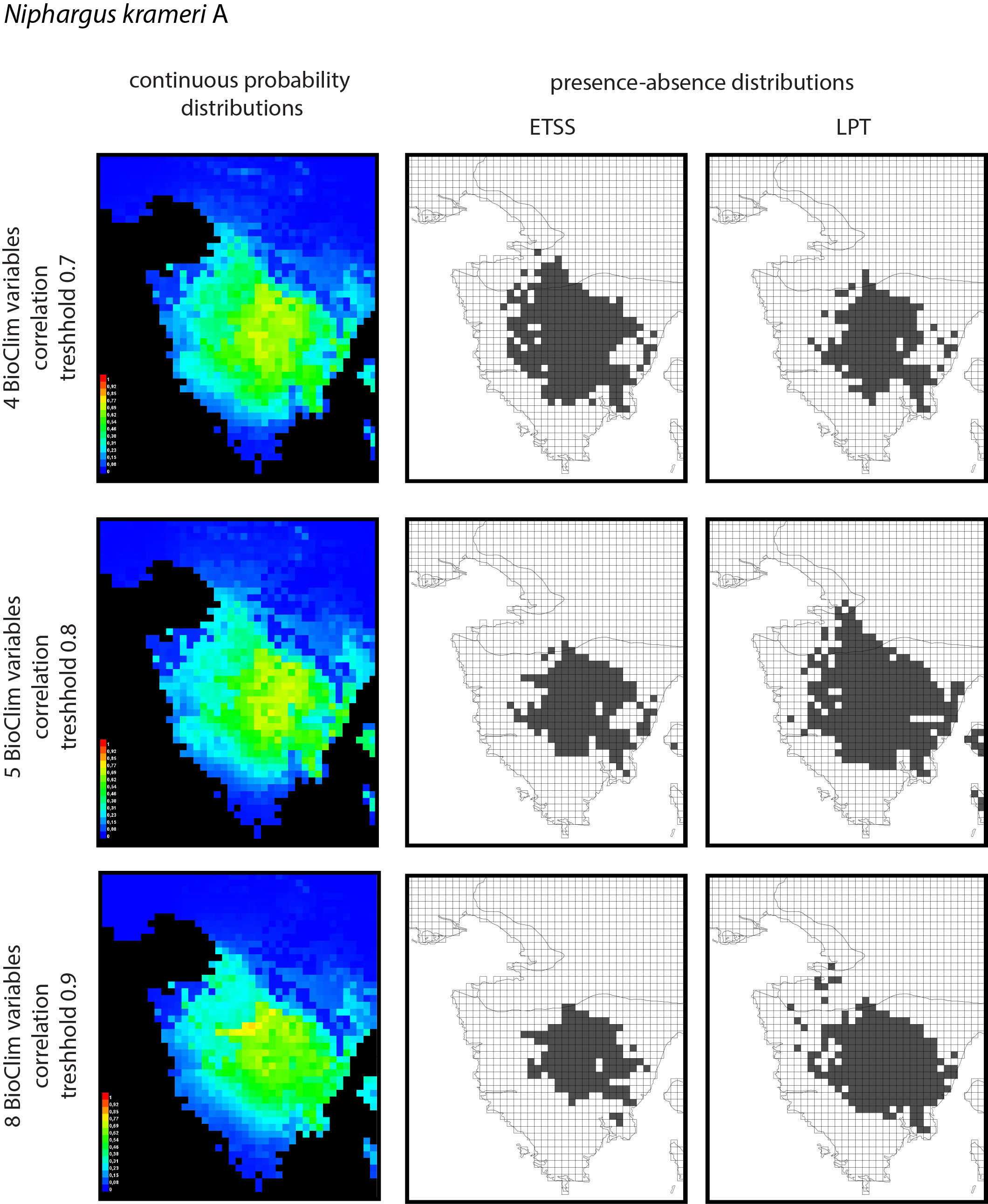


**S1 Fig.** Estimated distribution of *Niphargus krameri* A. Left column indicates continuous probability distributions, warm colors indicate high probability. Middle and right columns present presence-absence distributions using ETSS and LPT thresholds. Upper row is based on four, middle on five and lower on eight BioClim variables (see Table S1). Mean AUC values estimated from five subsets of original datasets are 0.851 +/- 0.039 (upper), 0.841 +/- 0.051 (middle), 0.868 +/- 0.052 (lower).
