# Supplementary material for: Morphologically Cryptic Amphipod Species Are “Ecological Clones” at Regional but Not at Local Scale: A Case Study of Four Niphargus Species
Source: PLoS One. 2015 Jul 30;10(7):e0134384. doi: 10.1371/journal.pone.0134384 (PMC4520478; doi:10.1371/journal.pone.0134384)
Supplement: S4 Table — (DOC) [file pone.0134384.s008.doc]

**Morphologically cryptic amphipod species are “ecological clones” at regional but not at local scale: a case study of four *Niphargus* species**

**Supporting Information S4 Table**

Žiga Fišer^1^, Florian Altermatt^2,3^, Valerija Zakšek^1^, Teja Knapič^4^, Cene Fišer^1^

^1^Department of Biology, Biotechnical Faculty, University of Ljubljana; Večna pot 111, SI-1001, Ljubljana, Slovenija.

^2^Department of Aquatic Ecology, Eawag: Swiss Federal Institute of Aquatic Science and Technology, Überlandstrasse 133, CH-8600 Dübendorf, Switzerland.

^3^Institute of Evolutionary Biology and Environmental Studies, University of Zurich

Winterthurerstr. 190, CH-8057 Zürich, Switzerland.

^4^Slovenian Museum of Natural History, Prešernova 20, SI - 1001 Ljubljana, Slovenija.

**S4 Table.** Evidence for competition inferred from presence-absence distributions corrected for bioclimatic niche envelope.

| **Sympatry^1^**  **(sp. 1-sp. 2)** | **Correlation threshold^2^** |  | **Species 1^3^** | **Species 2^4^** | **Syntopies^5^** | **N** | **M^6^** | **p-value^7^** |
| --- | --- | --- | --- | --- | --- | --- | --- | --- |
| NKA - NKB | 0.7 | obs. | 22 | 14 | 0 | 36 | 703 | **< 0.01** |
|  |  | exp. | 18.8505 | 6.27421 | 10.8753 |  |  |  |
| NKA - NKB | 0.8 | obs. | 25 | 19 | 0 | 44 | 1035 | **< 0.01** |
|  |  | exp. | 16.2941 | 13.0973 | 14.6085 |  |  |  |
| NKA - NKB | 0.9 | obs. | 24 | 11 | 0 | 35 | 666 | **< 0.01** |
|  |  | exp. | 20.8352 | 4.49127 | 9.6735 |  |  |  |
| NKA - NSA | 0.7 | obs. | 13 | 4 | 0 | 17 | 171 | **< 0.01** |
|  |  | exp. | 12.4328 | 1.01493 | 3.55224 |  |  |  |
| NKA - NSA | 0.8 | obs. | 12 | 4 | 0 | 16 | 153 | **< 0.01** |
|  |  | exp. | 10.4402 | 1.5444 | 4.01544 |  |  |  |
| NKA - NSA | 0.9 | obs. | 14 | 3 | 1 | 18 | 190 | **0.055** |
|  |  | exp. | 13.4552 | 0.95681 | 3.58804 |  |  |  |
| NKA - NSB | 0.7 | obs. | 5 | 25 | 3 | 33 | 595 | **0.034** |
|  |  | exp. | 1.85752 | 24.409 | 6.73351 |  |  |  |
| NKA - NSB | 0.8 | obs. | 6 | 49 | 3 | 58 | 1770 | **< 0.01** |
|  |  | exp. | 1.18427 | 49.1838 | 7.63196 |  |  |  |
| NKA - NSB | 0.9 | obs. | 5 | 11 | 3 | 19 | 210 | 0.249 |
|  |  | exp. | 2.71429 | 10.8571 | 5.42857 |  |  |  |
| NKB - NSA | 0.7 | obs. | 17 | 4 | 1 | 22 | 276 | **0.011** |
|  |  | exp. | 16.5114 | 1.14345 | 4.34511 |  |  |  |
| NKB - NSA | 0.8 | obs. | 14 | 4 | 1 | 19 | 210 | **0.027** |
|  |  | exp. | 13.4737 | 1.31579 | 4.21053 |  |  |  |
| NKB - NSA | 0.9 | obs. | 5 | 3 | 0 | 8 | 45 | 0.099 |
|  |  | exp. | 3.27869 | 2.09836 | 2.62295 |  |  |  |
| NKB - NSB | 0.7 | obs. | 38 | 128 | 15 | 181 | 16653 | **< 0.01** |
|  |  | exp. | 16.2584 | 120.483 | 44.2589 |  |  |  |
| NKB - NSB | 0.8 | obs. | 33 | 128 | 15 | 176 | 15753 | **< 0.01** |
|  |  | exp. | 13.6885 | 121.526 | 40.786 |  |  |  |
| NKB - NSB | 0.9 | obs. | 34 | 128 | 15 | 177 | 15931 | **< 0.01** |
|  |  | exp. | 14.0659 | 121.58 | 41.3538 |  |  |  |
| NSA - NSB | 0.7 | obs. | 2 | 6 | 0 | 8 | 45 | 0.12 |
|  |  | exp. | 0.91139 | 4.96203 | 2.12658 |  |  |  |
| NSA - NSB | 0.8 | obs. | 2 | 6 | 0 | 8 | 45 | 0.12 |
|  |  | exp. | 0.91139 | 4.96203 | 2.12658 |  |  |  |
| NSA - NSB | 0.9 | obs. | 1 | 2 | 0 | 3 | 10 | 0.534 |
|  |  | exp. | 0.42857 | 1.71429 | 0.85714 |  |  |  |

^1^ Sympatry of species pair in area of overlapping ranges as inferred by LPT binary threshold.

^2^ Correlation threshold defines the BioClim variables used in modeling (see S1 Table).

^3-5^ Observed and expected frequencies of species 1 and 2 when found alone and in syntopy.

^6^ Value of M statistic.

^7^ Probability that observed frequencies come from the same underlying distributions as expected frequencies.
