# Supplementary material for: Morphologically Cryptic Amphipod Species Are “Ecological Clones” at Regional but Not at Local Scale: A Case Study of Four Niphargus Species
Source: PLoS One. 2015 Jul 30;10(7):e0134384. doi: 10.1371/journal.pone.0134384 (PMC4520478; doi:10.1371/journal.pone.0134384)
Supplement: S5 Table — (DOC) [file pone.0134384.s009.doc]

**Morphologically cryptic amphipod species are “ecological clones” at regional but not at local scale: a case study of four *Niphargus* species**

**Supporting Information S5 Table**

Žiga Fišer^1^, Florian Altermatt^2,3^, Valerija Zakšek^1^, Teja Knapič^4^, Cene Fišer^1^

^1^Department of Biology, Biotechnical Faculty, University of Ljubljana; Večna pot 111, SI-1001, Ljubljana, Slovenija.

^2^Department of Aquatic Ecology, Eawag: Swiss Federal Institute of Aquatic Science and Technology, Überlandstrasse 133, CH-8600 Dübendorf, Switzerland.

^3^Institute of Evolutionary Biology and Environmental Studies, University of Zurich

Winterthurerstr. 190, CH-8057 Zürich, Switzerland.

^4^Slovenian Museum of Natural History, Prešernova 20, SI - 1001 Ljubljana, Slovenija.

**S5 Table.** Evidence for competition inferred from presence-absence distributions corrected for bioclimatic niche envelope and epi-hypogean spatial segregation.

| **Sympatry^1^**  **(sp. 1-sp. 2)** | **Correlation threshold^2^** |  | **Species 1^3^** | **Species 2^4^** | **Syntopies^5^** | **N** | **M^6^** | **p-value^7^** |
| --- | --- | --- | --- | --- | --- | --- | --- | --- |
| NKA - NKB | 0.7 | obs. | 11 | 5 | 0 | 16 | 153 | **0.007** |
|  |  | exp. | 8.68 | 2.58 | 4.74 |  |  |  |
| NKA - NKB | 0.8 | obs. | 12 | 9 | 0 | 21 | 253 | **< 0.001** |
|  |  | exp. | 6.45 | 7.57 | 6.99 |  |  |  |
| NKA - NKB | 0.9 | obs. | 12 | 4 | 0 | 16 | 153 | **0.009** |
|  |  | exp. | 10.06 | 1.75 | 4.19 |  |  |  |
| NKA - NSA | 0.7 | obs. | 13 | 4 | 0 | 17 | 171 | **0.004** |
|  |  | exp. | 12.43 | 1.01 | 3.55 |  |  |  |
| NKA - NSA | 0.8 | obs. | 12 | 4 | 0 | 16 | 153 | **0.008** |
|  |  | exp. | 10.44 | 1.54 | 4.02 |  |  |  |
| NKA - NSA | 0.9 | obs. | 14 | 3 | 1 | 18 | 190 | *0.055* |
|  |  | exp. | 13.46 | 0.96 | 3.59 |  |  |  |
| NKA - NSB | 0.7 | obs. | 5 | 25 | 3 | 33 | 595 | **0.034** |
|  |  | exp. | 1.86 | 24.41 | 6.73 |  |  |  |
| NKA - NSB | 0.8 | obs. | 6 | 49 | 3 | 58 | 1770 | **< 0.001** |
|  |  | exp. | 1.18 | 49.18 | 7.63 |  |  |  |
| NKA - NSB | 0.9 | obs. | 5 | 11 | 3 | 19 | 210 | 0.249 |
|  |  | exp. | 2.71 | 10.86 | 5.43 |  |  |  |
| NKB - NSA | 0.7 | obs. | 5 | 1 | 0 | 6 | 28 | 0.230 |
|  |  | exp. | 4.84 | 0.19 | 0.97 |  |  |  |
| NKB - NSA | 0.8 | obs. | 5 | 1 | 0 | 6 | 28 | 0.230 |
|  |  | exp. | 4.84 | 0.19 | 0.97 |  |  |  |
| NKB - NSA | 0.9 | obs. | 0 | 0 | 0 | 0 | 0 | / |
|  |  | exp. | 0.00 | 0.00 | 0.00 |  |  |  |
| NKB - NSB | 0.7 | obs. | 19 | 60 | 5 | 84 | 3655 | **< 0.001** |
|  |  | exp. | 7.61 | 55.79 | 20.60 |  |  |  |
| NKB - NSB | 0.8 | obs. | 18 | 60 | 5 | 83 | 3570 | **< 0.001** |
|  |  | exp. | 7.03 | 56.12 | 19.86 |  |  |  |
| NKB - NSB | 0.9 | obs. | 18 | 60 | 5 | 83 | 3570 | **< 0.001** |
|  |  | exp. | 7.03 | 56.12 | 19.86 |  |  |  |
| NSA - NSB | 0.7 | obs. | 2 | 6 | 0 | 8 | 45 | 0.12 |
|  |  | exp. | 0.91 | 4.96 | 2.13 |  |  |  |
| NSA - NSB | 0.8 | obs. | 2 | 6 | 0 | 8 | 45 | 0.12 |
|  |  | exp. | 0.91 | 4.96 | 2.13 |  |  |  |
| NSA - NSB | 0.9 | obs. | 1 | 2 | 0 | 3 | 10 | 0.534 |
|  |  | exp. | 0.43 | 1.71 | 0.86 |  |  |  |

^1^ Sympatry of species pair in area of overlapping ranges as inferred by LPT binary threshold.

^2^ Correlation threshold defines the BioClim variables used in modeling (see S1 Table).

^3-5^ Observed and expected frequencies of species 1 and 2 when found alone and in syntopy.

^6^ Value of M statistic.

^7^ Probability that observed frequencies come from the same underlying distributions as expected frequencies.
